# A 2-year prospective study of patient-relevant outcomes in patients operated on for knee osteoarthritis with tibial osteotomy

**DOI:** 10.1186/1471-2474-6-18

**Published:** 2005-04-05

**Authors:** Annette W-Dahl, Sören Toksvig-Larsen, Ewa M Roos

**Affiliations:** 1 Department of Orthopedics, Lund University Hospital, SE-221 85 Lund, Sweden

## Abstract

**Background:**

Tibial osteotomy is a treatment for younger and/or physically active patients suffering from uni-compartmental knee osteoarthritis. The open wedge osteotomy by the hemicallotasis technique includes the use of external fixation. The use of external fixation has several advantages, as early mobilization and the opportunity for optimal correction. However, the hemicallotasis technique has also been described as a cumbersome procedure for the patient. The aim of this study was to prospectively evaluate patient-relevant outcomes during the first 2 post-operative years. Especially the treatment period, during which external fixation was used, was closely monitored.

**Methods:**

In an uncontrolled study, fifty-eight consecutive patients, 30 men and 28 women (mean age 54 years) were operated on by the hemicallotasis technique were evaluated with the patient-relevant outcome measure Knee injury and Osteoarthritis Outcome Score (KOOS) preoperatively, during the treatment with external fixation, one week after removal of the external fixation, at 6 months, and at one and two years postoperatively.

**Results:**

At the 2-year postoperative follow-up, all subscales of the KOOS were improved (p < 0.001), mostly in pain (41–80 on a 0–100 worst to best scale) and knee-related quality of life (21–61 on a 0–100 worst to best scale), compared to the preoperative status. Significant improvements in pain and other symptoms, function of daily life and quality of life were seen already during the treatment period (mean 98 ± 18 days) with the external fixation. More demanding functions such as kneeling, squatting, jumping and running, were improved first after extraction of the external fixation device and the pins.

**Conclusion:**

Tibial osteotomy by the hemicallotasis technique yields large improvement in self-rated pain, function and quality of life, which persists over two years. Surprisingly, large improvements occurred already during the immediate post-operative period when the external fixation was still used.

## Background

**Figure 1:**
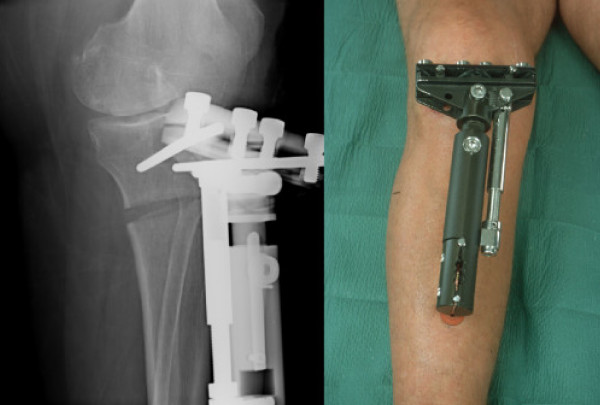
Hemicallotasis osteotomy a. After correction b. The Orthofix T-garche

Tibial osteotomy is a treatment for younger and/or physically active patients suffering from uni-compartmental knee osteoarthritis (OA) [1]. Traditional closed wedge osteotomy is regarded as the golden standard for osteotomy techniques. The closed wedge osteotomy is technically difficult and the degree of correction achieved may be unpredictable. In many cases the rehabilitation time and sick leave period is long [2]. Hemicallotasis osteotomy (HCO) is an open wedge technique, which implies a successive correction of the angle deformity of the knee postoperatively under radiographic control (Figure 1). The advantages of the HCO include an easy surgical technique and improved possibility to achieve the planned correction [3-5]. As the technique is based on the use of an external fixator just below the knee joint, early mobilization and daily living as well as recreational sports activities are possible [1,3]. HCO can be performed in medial and lateral knee OA. However, HCO has also been described as a cumbersome procedure for the patient as well as for the surgeon, due to frequent minor complications requiring frequent follow-ups [4,6]. Little is known from the patients' perspective regarding treatment with external fixation and the outcome of HCO. This information is valuable in helping patients make treatment choices and preparing them for the treatment period.

The purpose was, in a prospective study, to evaluate pain, function and quality of life during two years in patients operated on for knee OA with tibial osteotomy by the hemicallotasis technique. Especially the treatment period, during which external fixation was used, was closely monitored.

## Methods

### Patients

**Figure 2:**
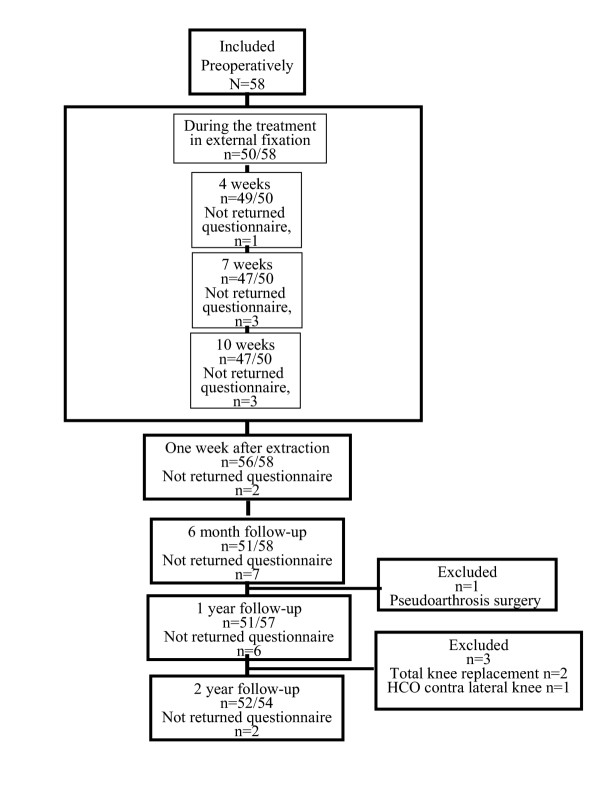
Flow chart of the study design.

58 consecutive patients (30 men and 28 women), mean age 54 years (36–69) were operated on for knee OA by HCO at the Department of Orthopaedics, Lund University Hospital, Sweden were included in a uncontrolled study. Patient characteristics are given in Table 1 and a flow chart of the study design is given in Figure 2.

**Table 1:** Patient characteristics

	All N = 58	Men n = 30	Women n = 28
Age *mean (years)*	54	54	55
*SD*	7	8.7	4
			
BMI *mean*	29	28.7	29.5
*SD*	4.5	4	5
			
Medial OA *(n)*	49	27	22
Pre HKA *mean (degrees*)	170	170	171
*SD*	4.5	5	3.5
			
Lateral OA *(n)*	9	3	6
Pre HKA *median (degrees*)	189	188	189.5
*IQR*	184–194	184–190	184–194
			
Known knee trauma *(n)*	27	16	11
*mean age (years)*	51	50	53
*SD*	7	8	5
			
Not known knee trauma *(n)*	28	12	16
*mean age (years)*	58	58	56
*SD*	5	7	4
			
Physically active earlier in life *(n)*	45	25	20
Level of physical activity *(n)*			
recreational	23	13	10
competitive	12	11	1
			
Joint loading in sport activity *(n)*^*a*^			
low	21	7	14
medium	12	6	6
high	12	12	0
			
Physically active the year prior surgery *(n)*	22	14	8
Smokers *(n)*	14	6	8
			
Working *(n)*	51	26	25
Full time (n)	44	26	18
Part time (n)	7	0	7
Disability pension *(n)*	3	0	3
Retired *(n)*	4	4	0

BMI = Body Mass Index, OA = Osteoarthritis, Pre HKA = Preoperative radiographic Hip-Knee-Ankle angle, IQR = Inter Quartile Range ^*a*^according to Buckwalter & Lane [11].

### Preoperative information

When a patient was recommended HCO, written and verbal information was given by a specially trained nurse in our outpatient clinic for patients treated with external fixation.

### Operation

Four conical pins were inserted, 2 hydroxyapatite (HA)-coated pins in the metaphyseal bone and 2 standard pins (Orthofix^® ^Bussolengo, Italy) in the diaphyseal bone. A 5 – 7-cm longitudinal skin incision for the osteotomy was placed ventral to the tibial tuberosity. After a transverse incision of the periosteum, an osteotomy was performed. The periosteum was sutured and the wound was closed. After draping the pin sites and the incision, the fixator (Orthofix^® ^T-garche) was mounted. For valgus deformity, the surgical procedure was the same except that a fibulotomy was performed 10 – 15 cm below the head of the fibula [7]. The surgical procedure took about 30 minutes. In most cases the patients were discharged the same day.

### Postoperative treatment

Once a week during the treatment period the patients visited the outpatient clinic for pin site care, initiation and follow-up of the correction. The correction started 7–10 days postoperatively. The patient made the correction by adjusting one quarter of a turn 4 times per day on a distractor placed at the external fixator, the first turn in the morning and the last turn, not later than 5 pm to avoid pain at night. The patients got instructions how to slow down the correction in case of unacceptable pain. When the desired correction, 4° valgus for the varus knee and 0–2° varus for the valgus knee, was achieved as determined by radiographic hip-knee-ankle angle (HKA-angle) of the knee, the instrument was locked. Eight weeks postoperatively the instrument was dynamised, unlocking the fixation partly to allow micro movement of the osteotomy at weight bearing, in order to stimulate bone healing. About 12 weeks postoperatively, the first healing control by radiography and ultrasound examination was done. If the osteotomy healing was satisfying the patient made a weight bearing test; i.e., walking (with or without crutches) for an extended period of time varying from some hours to some days without the instrument but still with the pins in situ. If no symptoms arouse, the pins were removed at the out patient clinic. If the patient developed symptoms, the fixation was applied again for another 2–4 weeks.

### Postoperative exercise

Exercise and physical therapy were recommended to the patients when receiving the preoperative information. Immediately after surgery the patients were informed by a physiotherapist. Full weight-bearing and free mobilization was allowed postoperatively. Physiotherapy was prescribed individually and related to the needs for each patient. The patient and physiotherapist decided jointly the length of the rehabilitation period needed.

### Outcome measure

KOOS (Knee injury and Osteoarthritis Outcome Score) [8,9] was used as the primary outcome measure. KOOS is a 42-item self-administrated knee-specific questionnaire based on the WOMAC Index [10]. KOOS was developed to be used for short- and long-term follow-up studies of knee injury and knee OA, and comprises five subscales: Pain, Symptoms, Activities of Daily Life (ADL), Sports and Recreation Function (Sport/Rec) and knee-related Quality of Life (QOL). Standardized answer options are given (5 Likert boxes), and each question gets a score from 0 to 4. A score from 0 to 100 is calculated for each subscale; 100 represent the best results. The KOOS can be downloaded from . A difference of 10 points was considered a clinically significant difference [11].

Physical activity level was categorized as competitive sports, recreational sports or no sport at all. Regular walks were classified as recreational sports. At baseline, information was obtained regarding physical activity level earlier in life and during the year prior to surgery. According to Buckwalter and Lane [12], the activities performed were divided into high (e.g. team handball, high mileage running, football-soccer, rugby, water skiing), medium (e.g. canoeing, horse-riding, downhill skiing, basketball), and low (e.g. swimming, golf, cycling, walking) joint loading. The patient's previously known knee injuries (meniscus and/or anterior cruciate ligament injury) were documented. The patient's current working status (working full time or part-time, disability pension or retired) was documented.

Complications such as a loose pin, delayed healing, pseudoarthrosis, septic arthritis, interrupted treatment, deep venous thrombosis and nervous injury were recorded. A loose pin was defined as a pin, which could be removed by hand without use of a wrench. Pin site infection was classified according to the Checketts- Otterburns classification [13]. Grades 1–3 are classified as minor infections and respond to proper treatment and external fixation can be continued. Major infections include soft tissue and/or bone and the treatment by external fixation may be interrupted. Grade 1 infections were not classified, as a complication as this grade of infection did not required any antibiotic or surgical treatment. Paracetamol and Tramadol were prescribed as analgesics. At the weekly visit, the patients reported the analgesic consumption during the previous week to the specialist nurse at the out patient clinic.

The patients were defined as non-smokers if they, at the preoperative visit, reported that they never had smoked or stopped smoking since more than 6 month. The radiographic hip-knee-ankle (HKA)-angle was determined after the correction was performed and at the 2-year follow-up. A HKA-angle of <180 degrees was indicated varus alignment of the knee.

### Design

The KOOS questionnaire was distributed to the 58 patients by mail and returned in a pre-stamped envelope.

The patients were asked to answer the Swedish version of KOOS LK 1.0, preoperatively, one week after removal of the external fixator and the pins, 6 months, one year and two years postoperatively. 50 of the patients were also asked to answer the KOOS during the treatment with external fixator at 4, 7 and 10 weeks postoperatively. The evaluation time points during the treatment with external fixator reflected the different phases of the treatment: a) correction, b) after correction, when the instrument was locked and c) the final stage of the treatment.

The study was approved by the Ethics Committee at the Medical Faculty, Lund University.

### Statistical analysis

The underlying data obtained from questionnaires such as the KOOS are ordinal, which implies the use of non-parametric statistics. However, means and standard deviations are often given instead of medians and inter-quartile ranges for this type of questionnaire data. Postoperative change over time were assessed by Friedman's test, and changes between two postoperative measurements were assessed by Wilcoxon's rang test for each of the five KOOS subscales. The level of significance was set to p 0.05. The influence of 7 potential predictor variables (age, BMI, sex, acceptable HKA-angle at the 2-year follow-up, smoking and complications) on improvements at the 2 year follow-up compared to baseline in pain and knee-related QOL was analyzed by means of linear regression analysis. First, the influence of each potential predictor was assessed in simple regression analysis. Those predictors that implied p < 0.20 were considered further in a multivariate regression analysis.

## Results

52/58 patients (90 %) were evaluated at the 2-year follow-up (Fig 2). Due to geographic reasons, 14 patients (8 medial OA, 6 lateral OA) were not followed up by radiography including the HKA-angle at 2 years. The mean correction time was 24 (SD 12) days and the mean time in external fixation was 98 (SD 18) days. The desired HKA-angle was achieved in 57/58 patients (septic arthritis, n = 1) after the correction phase. The mean HKA-angle after correction phase was 183 ± 1 degrees for the varus knees (n = 49) and 180 ± 2 degrees for the valgus knees (n = 9). 2-year postoperatively the mean HKA-angle was 182 ± 3.5 degrees in the varus knees and mean 177 ± 3 degrees in the valgus knees.

### Complications

During the treatment period in external fixation, 16/58 patients had complications (Table 2). 1/6 patients with a loose pin at the time of removal of the fixator and pins, had clinical pin site infection grade 2 according to the Checketts-Otterburns classification [13] at some point during the treatment. One patient developed septic arthritis and did not achieve the desired correction and had revision surgery by total knee replacement 20 month after the HCO Two patients developed deep venous thrombosis and were successfully treated by Warfarin. One patient with delayed healing developed pseudoarthrosis and healed after additional surgery. One patient lost the achieved correction after extraction of the fixator and pins but required no additional surgery.

**Table 2:** Complications during the 2 years follow-up.

Complication	Patients (n)
Pin site infection grade 2 [13]	5
Loose pin at removal of fixator/pins	6
Septic arthritis	1
Deep venous thrombosis	2
Loss of correction	1
Pseudoarthrosis	1

### Work level

At the 2-year follow-up 40/52 (77%) patients were working. 10 of the patients (equal number of men and women) had decreased their time spent working compared to preoperatively (Table 1). Of these, 2 patients had received disability pension due to other reasons (fibromyalgia, n = 1 and one low back pain, n = 1) and one patient was on sick leave due to surgery in the contra lateral knee). Two patients had retired during the follow-up period.

### Improvement during the 2 year-follow-up

At the 2-year follow-up, the patients reported a mean pain reduction of 40/100 points compared to baseline (p < 0.001). Parallel improvement was seen in function and quality of life as determined by the KOOS (p < 0.001) (Table 3). Since 6/58 patients were unavailable for 2 year-follow-up an ITT (Intension To Treat) analysis using the last obtained KOOS value was performed as a substitute for the missing 2 year follow-up value. The ITT analyses yield very similar results.

**Table 3:** Mean scores of the KOOS preoperatively to two years postoperatively

	Treatment period				Follow-ups			
KOOS subscale (mean, SD)	Preop (N = 58)	4 weeks (N = 49)	7 weeks (N = 47)	10 weeks (N = 47)	One week after extraction (N = 56)	6 month (N = 51)	one year (N = 51)	two years (N = 52)

Pain	41	51	59	62	71	74	75	80
	17	22	22	20	21	20	20	20
								
Symptom	50	55	61	63	68	74	75	80
	18	19	19	19	19	20	20	20
								
Activities of daily life	48	49	57	62	71	75	79	80
	19	20	19	16	20	21	20	19
								
Sport and recreation function	9	2	6	5	11	20	30	29
	12	5	10	9	15	24	29	28
								
Knee related quality of life	21	24	33	32	43	52	56	61
	14	19	21	18	22	25	27	25

KOOS = Knee injury and Osteoarthritis Outcome Score, 0–100 worst to best scale.

### Improvement during the treatment with external fixation

**Figure 3:**
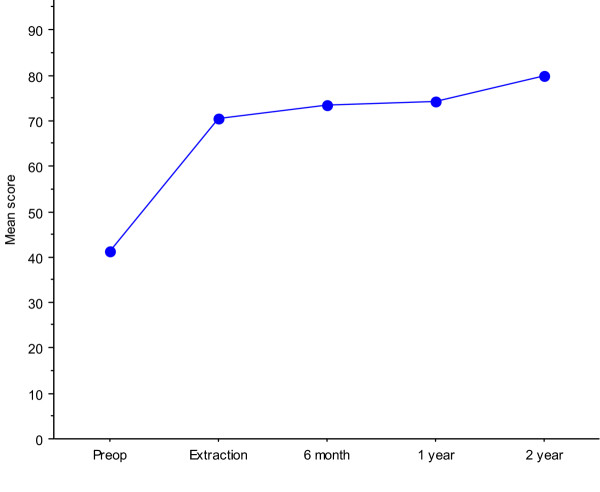
KOOS Pain mean scores (± 95%CI) over time (0–100, worst to best).

**Figure 4:**
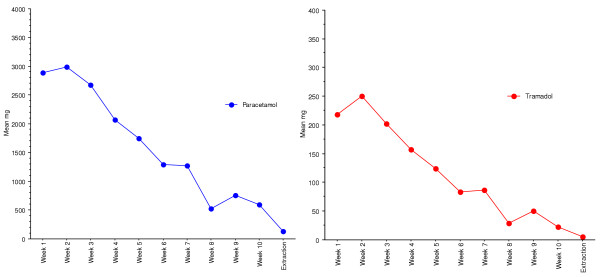
Average analgesic consumption during the treatment period (n = 50).

The major improvements in pain and ADL function were obtained during the treatment with the external fixator, thereafter further but smaller improvements were seen until the 2-year follow-up as exemplified in Figure 3. Already at four weeks postoperatively, a significant pain reduction (p = 0.02) was seen (Table 3). There was also a significant reduction of the analgesic consumption at week 4, paracetamol (p < 0.001) and tramadol (p < 0.001) compared to the first postoperative week (Fig 4.). Reductions of the percentages of patients reporting both activity-related pain and pain at rest were seen. Preoperatively, 90% of the patients reported moderate to extreme pain during walking (activity-related pain, question 5 in KOOS subscale pain) compared to 70 % at week 4, 55% at week 7 and 36% at extraction of the external fixation and pins. Preoperatively, 55% of the patients reported moderate to extreme pain at night (pain at rest, question 7 in KOOS subscale pain) compared to 55% at week 4, 38% at week 7 and 8% at extraction of the external fixation and pins.

At 7 weeks a significant improvement compared to preoperatively was seen with regard to symptoms (p = 0.002), activities of daily living (p = 0.003) and knee-related quality of life (p < 0.0001) (Table 3).

### Improvements after removal of the external fixation

Compared to one week after removal of the external fixation, significant improvements were seen in knee-related quality of life (p = 0.005) at the 6-month follow-up. At the one-year follow-up, improvement was seen in sport and recreation function (p = 0.0001) which assessed more demanding functions such as kneeling, squatting, jumping and running. At the 2-year follow-up improvement were seen in the subscales pain (p = 0.01), symptom (p = 0.02) and ADL (p = 0.02) (Table 3).

### Predictors of poor improvement

Worse preoperative pain and complications were predictors of poor improvement in the KOOS subscale pain (Table 4). Age, BMI, sex, acceptable HKA-angle two years postoperatively and smoking were not predictors for poor improvement in pain. Performing the same analysis for poor improvement in the KOOS subscale knee-related QOL, there were no predictors of poor improvement.

**Table 4:** Linear regression preformed to determind predictors of poor changes preoperatively to the 2 years follow-up of the KOOS subscales pain.

	Univeriate analysis					Multivariate analysisII		
	
Predictor Variable	N^a^	C^b^	(95% CI)	p^c^	R2 adj^d^	C^b^	(95% CI)	p^c^
Age (year)	52	0.27	(-0.62–1.17)	0.54	0%		Not included	
BMI (kg/m2)	48	0.62	(-0.89–2.12)	0.41	0%		Not included	
Preop pain (0–100)	52	'-0.61	(-0.92–-0.29)	0.0003	21%	'-0.6	(-0.9–-0.2,8)	0.0004
Sex	52	1.42	(-10.77–13.6)	0.82	0%		Not included	
0: female	26							
1: male	26							
Acceptable 2 year HKA-angle	42	8.78	(-12.81–30.38)	0.41	0%		Not included	
0: no	7							
1: yes	37							
Smoker	52	'-0.35	(-15.27–14.56)	0.96	0%		Not included	
0: no	40							
1: yes	12							
Complication	51	'-15.8	(-16.6–7.94)	0.06	0.5%	'-14.7	(-29.4–0.08)	0.05
0: no	43							
1: yes	8							

^a^Number of patients included

^b^Estimated change of changes in preoperative until 2 years postoperative pain per unit change predictor variable; 95% confidence interval (CI) within paranthesis.

^c^*P *value for effect of predictor variable.

^c^Fraction of changes of preopertive until 2 years postoperative pain variance explaind by each predictor (adjusted value).

## Discussion

This study shows large improvements in self-rated pain, function and quality of life at 2 years for patients operated on for knee OA by tibial osteotomy using the hemicallotasis technique. Surprisingly, substantial improvements were seen already during the immediate postoperative period when the external fixation was still used.

To our knowledge, this follow-up study is the first evaluating the patients' perspective of the HCO including the treatment period. Clinical scores have been used by Magyar et al in a randomized study comparing close wedge osteotomy and HCO, and by Gerdhem et al when evaluating the HCO [2,6]. Both studies showed significant improvements and good to excellent results as evaluated by the Hospital for Special Surgery Score (HSS), at 2 years and at 12 to 28 months respectively. Our results are in line with these previous reports.

Magyar et al [2] found no differences in clinical scores between two methods of high tibial osteotomy and remarked that the clinical scores used, seemed to be too blunt to detect the differences in younger active patients. For this reason we choose an outcome measure validated for younger or physically active subjects with knee OA. The Knee injury and Osteoarthritis Outcome Score (KOOS) evaluates pain, other symptoms and activities of daily living but also includes sport and recreational activities and quality of life, dimensions that have been shown to be more sensitive in younger and/or physically active with knee OA than the more commonly used Western Ontario and MacMaster Universities Osteoarthritis Index (WOMAC) [14]. The patients filled in the questionnaires themselves in their homes. In studies using this administration mode and frequent follow-ups a high number of dropouts could be expected. In the present study the 2-year follow-up rate was 90%, which must be considered high.

Most of the improvements in all subscales of the KOOS, except for sport/recreational function, were obtained during the treatment period, when using the external fixation. The KOOS questionnaire was sensitive enough to detect significant changes over just a few weeks. Four weeks postoperatively, pain related to the correction was common, but the pain gradually diminished. Seven weeks postoperatively, the correction was completed and the external fixator was locked. When visiting the outpatient clinic, the patients told that they felt improvements almost day by day, as confirmed by the improvements in all KOOS subscales except for sports/recreation function. During the later part of the treatment, patients gained more knee stability and gradually decreased the use of crutches, but were still prevented from certain activities due to the external fixator. This clinical improvement was detected by the KOOS, especially the subscale ADL. One week after finishing the treatment with external fixator knee-related QOL was further improved, probably due to the extraction of the fixator and the pins. The similar decrease of number of patients with moderate to extreme pain during walking and at rest reported during the treatment in external fixation, indicate that the treatment by the HCO effected activity-induced pain as well as pain at rest.

Most probably the early pain reduction seen is due to the gradually corrected alignment of the leg. Alternative explanations to the early improvements seen include decrease of intraosseous pressure [15,16].

The gender distribution in our study was almost even reflecting that knee injuries, and thus post-traumatic OA due to knee injuries, are more common in men [17], whereas elderly women more commonly have OA. The patients with known knee injury in this study were mostly men (28%). They were, on average, 8 years younger than men without known knee injury, and had performed more high/medium joint-loading sport activities. This reflects that patients with joint injury have an increased risk of developing knee OA requiring surgery [17-19].

Patients having tibial osteotomy in the current study and patients having knee arthroplasty in a previous study report similar preoperative pain and function [20]. This is remarkable, taking into account the tibial osteotomy patient being on average 17 years younger. It should also be noted that patients developing OA at a younger age often have high demands of their knee function in both working life and leisure time.

Long-term results have been shown to depend on the achieved correction of the healed osteotomy [21-24]. 57/58 patients in our study achieved the intended correction. The mean HKA-angle at the 2-year follow-up was acceptable in 38/52 patients. These results are comparable or even better than studies with similar evaluation period [2,5,6].

As a predictor for poor improvements in pain over time, worse preoperative pain accounted for 21% of the variance. This is a factor that should be taken into consideration when selecting patients for high tibial osteotomy. This may indicate that operating earlier would give a better result as also discussed for total joint replacement [25].

Ten patients had complications and not unexpectedly, these complications were predictors of poor improvement in pain over time.

## Conclusion

Our study showed that tibial osteotomy by the hemicallotasis technique yields large improvement in self-rated pain, function, and quality of life, which persists over two years. Surprisingly, most improvements were seen already during the immediate post-operative period when the external fixation was still used. This new knowledge should be incorporated in the information to the patients to help them make treatment choices regarding knee OA and in the pre-operative information of the hemicallotasis technique.

## Competing interests

The author(s) declare that they have no competing interests.

## Authors' contributions

AWD, STL and ER designed the study. AWD collected the data, analyzed the data and drafted the manuscript. STL and ER revised the manuscript. All three authors read and approved of the final manuscript.

## Pre-publication history

The pre-publication history for this paper can be accessed here:


